# Automated Tracking of Whiskers in Videos of Head Fixed Rodents

**DOI:** 10.1371/journal.pcbi.1002591

**Published:** 2012-07-05

**Authors:** Nathan G. Clack, Daniel H. O'Connor, Daniel Huber, Leopoldo Petreanu, Andrew Hires, Simon Peron, Karel Svoboda, Eugene W. Myers

**Affiliations:** Janelia Farm Research Campus, Howard Hughes Medical Institute, Ashburn, Virginia, United States of America; University of California, San Diego, United States of America

## Abstract

We have developed software for fully automated tracking of vibrissae (whiskers) in high-speed videos (>500 Hz) of head-fixed, behaving rodents trimmed to a single row of whiskers. Performance was assessed against a manually curated dataset consisting of 1.32 million video frames comprising 4.5 million whisker traces. The current implementation detects whiskers with a recall of 99.998% and identifies individual whiskers with 99.997% accuracy. The average processing rate for these images was 8 Mpx/s/cpu (2.6 GHz Intel Core2, 2 GB RAM). This translates to 35 processed frames per second for a 640 px×352 px video of 4 whiskers. The speed and accuracy achieved enables quantitative behavioral studies where the analysis of millions of video frames is required. We used the software to analyze the evolving whisking strategies as mice learned a whisker-based detection task over the course of 6 days (8148 trials, 25 million frames) and measure the forces at the sensory follicle that most underlie haptic perception.

“This is a *PLoS Computational Biology* Software article.”

## Introduction

Rats and mice move their large whiskers (vibrissae), typically in a rhythmic pattern, to locate object features [1], [2], or to identify textures and objects [3], [4]. Whisker movements in turn are influenced by touch [1], [5]. The whisker system is a powerful model for studying the principles underlying sensorimotor integration and active somatosensation [6]. Critical to any mechanistic study at the level of neurons is the quantitative analysis of behavior. Whisker movements have been measured in a variety of ways. Electromyograms, recorded from the facial muscles, correlate with whisker movements [7]. This invasive method does not report whisker position and shape per se and is complimentary to whisker tracking. Imaging individual labeled whiskers, for example by gluing a high-contrast particle on the whisker [8], provides the position of the marked whisker, but does not reveal whisker shape. In addition, the particle will change the whisker mass and stiffness and thereby perturb whisker dynamics. Monitoring a single point along the whisker using linescan imaging [9], or a linear light sheet [10], can provide high-speed information about whisker position, but only at a single line of intersection.

High-speed (>500 Hz) videography is a non-invasive method for measuring whisker movements and forces acting on whiskers and yields nearly complete information about whiskers during behavior [1], [11]–[15]. The position of the whisker base with respect to the face reveals the motor programs underlying behavior. The deformation of whisker shape by touch can be used to extract the forces felt by the mouse sensory follicles [16], [17]. However, high-speed videography brings its own technical challenges. The large number of images required makes manual analysis impossible for more than a few seconds of video. Comprehensive studies require fully automated analysis. In addition, extraction of motions and touch forces demands accurate measurement of whisker shape, often with sub-pixel precision, and identification of rapidly moving whiskers across time. Finally, the large volume of video data potentially places severe demands even on advanced computational infrastructures, making efficient algorithms necessary.

Tracking whiskers is challenging. Whiskers can move at high speeds [12] and in complex patterns [1], [18]. Adjacent whiskers can have distinct trajectories. Moreover, whiskers are thin hairs (e.g. mouse whiskers taper to a thickness of a few micrometers) and thus provide only limited contrast in imaging experiments.

To address these challenges, we have developed software for tracking a single row of whiskers in a fully automated fashion. Over a manually curated database of 400 video sequences of head fixed mice (1.32×10^6^ cumulative images, 4.5×10^6^ traced whiskers, 8 mice), whiskers were correctly detected and identified with an accuracy of 99.997% (1 error per 3×10^4^ traced whiskers). In other whisker tracking systems, models constraining possible motions and whisker shapes have been used to aid tracking. In contrast, our approach uses statistics gleaned from the video itself to estimate the most likely identity of each traced object that maintains the expected order of whiskers along the face. As a result, the shapes of highly strained whiskers (curvature>0.25/mm) can be traced with sub-pixel accuracy, and tracking is faithful despite occasional fast motion (deflections >10,000 degrees/s).

Our method consists of two steps performed in succession: tracing and linking. Tracing produces a set of piecewise linear curves that represent whisker-like objects for each individual image in a video. Each image is analyzed independently. The linking algorithm is then applied to determine the identity of each traced curve. The trajectory of a whisker is described by collecting curves with the same identity throughout the video. Importantly, once a faithful tracing is completed, the original voluminous image data set is no longer required for downstream processing.

Once tracing is complete, a small set of features is tabulated for each curve. Based on these features, a heuristic is used to make an initial guess as to the identity of curves in a set of images where it works well. These initial identifications are then used to get a statistical description of the shapes and motions of whiskers. These statistics are then used to compute a final optimal labeling for each traced curve subject to the constraint that whiskers are ordered along the face.

**Figure 1:**
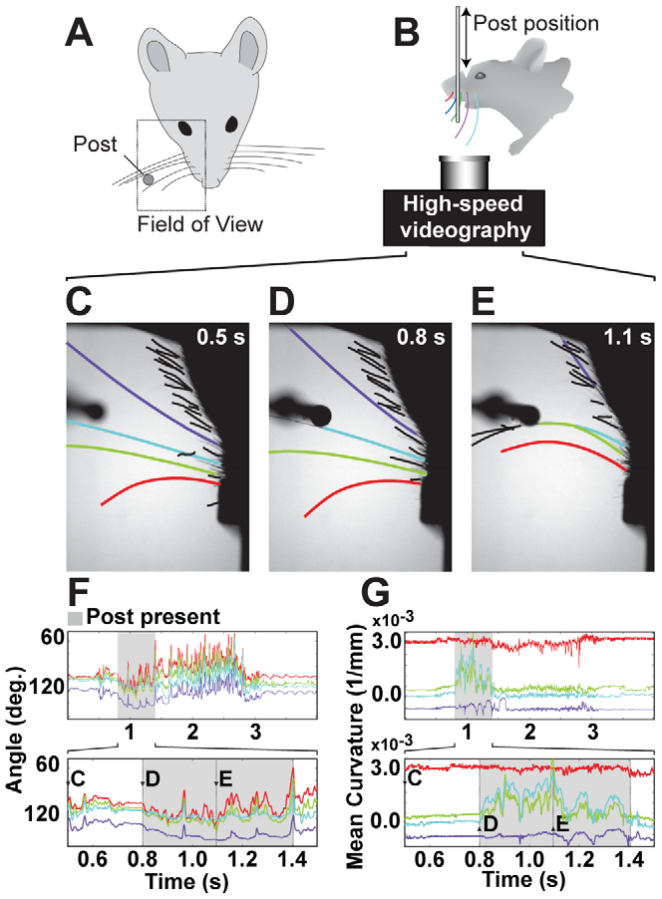
Tracking whiskers from high-speed (500 Hz) videos during an object detection task. (A) A typical field of view. (B) Typical imaging configuration. (C–G) Automated results of tracing and linking. (C) Facial hairs and whiskers are traced in each video frame and then identified by a separate tracking step. (D) A whisker (blue) touches the pole. (E) Two whiskers (blue & green) are bent by the pole. The most posterior whisker is strongly retracted so that only a small segment is visible. (F) Tracking measures whisker orientation, such as the angle at base. (G) Tracking measures whisker shape, such as mean curvature, which can be observed over time. Changes in curvature allow the calculation of forces acting on the whisker follicle [16].

We validated this approach using manually curated video acquired from a head-fixed, whisker-dependant object localization task [1]. In these experiments the field of view contains a single row of partially and wholly imaged whiskers as well as the stimulus object (a thin pole, normal to the imaging plane) (Figure 1A). The camera is positioned below the whiskers and illuminated from above (Figure 1B) producing silhouetted views of the whiskers (Figure 1C–D). Automated tracing is robust to occlusions introduced by the stimulus and to whisker crossings. Tracing yields curves for both whiskers and facial hairs (Figure 1C–D). Linking is used to uniquely identify each whisker so features such as angle of deflection (Figure 1F) and curvature (Figure 1G) can be extracted as time-series.

## Design and Implementation

### Tracing

The process of tracing the backbone of a whisker consists of three phases: initiation, extension, and termination. Tracing starts by using the angle and position estimated at the highest scoring candidate initiation site. As a curve is extended, initiation sites that fall under the traced curve are removed from consideration. When none remain, potentially duplicate curves are resolved.

Before any analysis, images were preprocessed to remove imaging artifacts specific to particular cameras (Text S1, Figure S1). Tracing begins by searching the image for candidate initiation sites. These are found by performing a pixel-level segmentation isolating locally line-like features in the image. With each segmented pixel, a score and an angle are computed. This is an optimization step; it is possible to initiate tracing at any pixel in an image. However, tracing is relatively expensive, and, by filtering out unproductive initiation sites, computation can be focused on the small number of relevant pixels. On the other hand, the filtration threshold should be set conservatively so that every whisker is sure to have at least one initiation site. Parameters were set by examining results over a small set of 10 images but worked well over the entire data set of over 1 million images. Typically, 50–100 candidate sites were found per whisker with 10–20 false positives per image.

**Figure 2:**
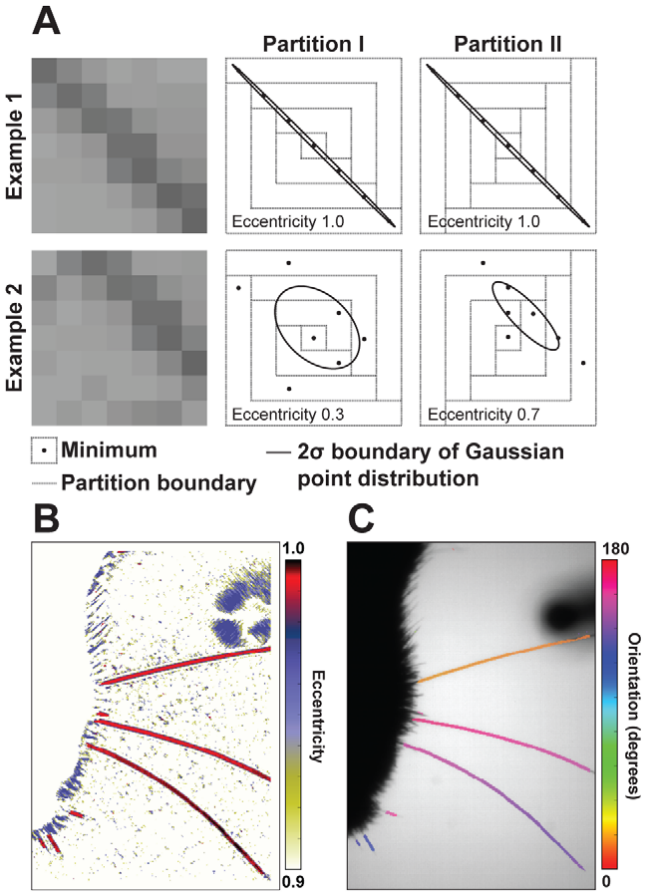
Detecting whiskers. (A) Lines passing through a 7×7 pixel box are detected by finding the location of intensity minima (black dots) in regions from two partitions. The eccentricity of the best Gaussian fit (indicated by ellipses) to these points is used to score salience. (B) The score computed at each point in an image. (C) For high scoring seeds (eccentricity>0.95), the orientation of the line is indicated by the major axis of the ellipse. Whisker tracing is initiated at these sites using the measured angle.

A variety of methods have been developed to detect interest points in natural images [19], and the Hough transform is conventionally used to find linear features. However, given the high contrast and controlled imaging conditions used to acquire data (Text S1), we developed a linear time algorithm, summarized in Figure 2, that requires only local image information and yields an estimate of salience and line orientation in a single pass.

First, a 7×7 square box is centered on a pixel of interest (Figure 2A). The box is divided into two partitions and the position of the intensity minima found in each subset of the two partitions is found. These are designed to ensure that when a whisker passes through the box, one partition will preferentially collect minima along the whisker backbone, and, as a result, the position of those minima will be linearly correlated. For each partition, principle components are computed over the covariance of the collected positions. The principle components describe the major and minor axis of an ellipse. The partition with the highest eccentricity is chosen, and the eccentricity is used to score how line-like the image is at the queried point (Figure 2B). The orientation of the major axis is used to estimate the direction of the line (Figure 2C, Video S1).

One advantage of this approach is that the entire image need not be queried for line-like objects. For example, in the data analyzed here, whiskers span hundreds of pixels. A grid of lines spaced appropriately (50 px; this is an adjustable parameter) will cross each whisker at least once. Restricting the search for line-like features to this grid greatly reduces the amount of time required for this step. Additionally, since the algorithm relies on local minima, demands on background subtraction and other preprocessing is minimal. The structure of the partitions ensures that lines in any orientation are detected. Greater than 80% of the pixels that could be used to start tracing a whisker (typically 1000 per whisker over the full image) are detected using an eccentricity threshold of 0.95; only 1 is required to fully trace a whisker. This detector systematically avoids potential starting locations near the face, near occlusions or where whiskers cross or nearly touch.

**Figure 3:**
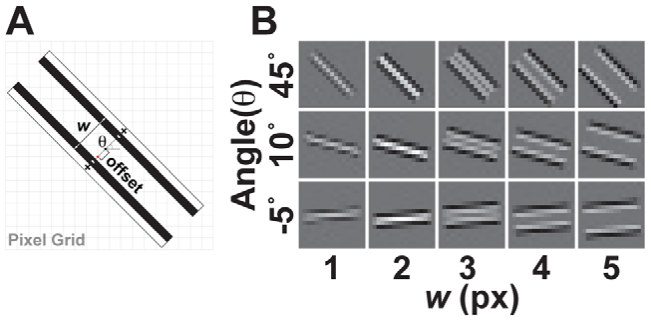
Parameterized line detector. (A) Two parallel step edge detectors are separated by a distance, *w*, and oriented by an angle, *θ* about a center point. The center point (black dot) is determined by a sub-pixel displacement from a pixel anchor (red dot). (B) The line detector is computed for discrete values of *w*, *θ*, and *offset*.

Curves are bidirectionally extended from candidate initiation sites by performing a small step (1 px) along the measured direction, and repeating until one of several criteria is met. The sub-pixel position and tangent angle are estimated by optimizing the correlation between an oriented line detector that is parameterized as a function of sub-pixel position, angle and width (Figure 3).

The line detector is designed based on modeling the intensity profile of a whisker as a rectangular valley in the image with variable position, width and angle. The center of the whisker is estimated with sub-pixel precision by finding a position that minimizes the Laplacian of the correlation between the model and the image [20]. The line detector approximates the Laplacian of the model. It consists of two rectangular parallel step-edge detectors (20×1 px) spaced by the detector width (Figure 3A). The length was chosen to match the distance over which highly curved whiskers remain approximately linear. The correlation at a given point is evaluated using a pixel representation of the detector that is computed by evaluating the area integral of the detector over the square domain of each pixel (Figure 3B). The value of the correlation at that position is then the dot product between pixels in the image and pixels in the evaluated detector. For efficiency, pixel representations of the detector are pre-tabulated for discrete values of the sub-pixel position (0.1 px), width (0.2 px), and angle (2.5 degree precision). Optimizing the width of the detector was important for reliable tracing of whiskers of different width and for reliable tracing of the very tips of whiskers.

**Figure 4:**
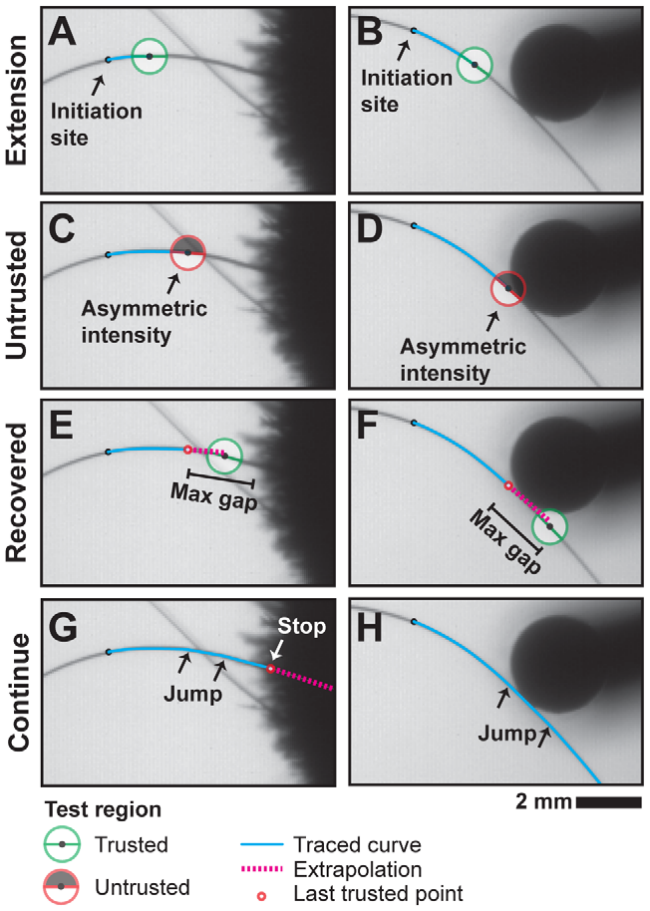
Tracing illustrated for cases with whisker crossing and pole contact. (A,B) As a curve is extended from an initiation point, a local test region within the image is queried at each step to detect cases where the line detector may be unreliable. This happens near (C) crossing whiskers and (D) whisker-pole contacts. (E,F) When such a cases are encountered, the curve is linearly extended from the last trusted point, up to a threshold distance. (G,H) If all tests are satisfied at one of the points, a line segment is used to jump the gap and normal tracing is resumed. Otherwise, the trace is terminated at the last trusted point.

Tracing is stopped if any one of several criteria indicates that the optimization procedure cannot be trusted to yield accurate results. This is necessary to handle cases where whiskers were partially occluded, such as when whiskers crossed or contacted an obstructing object, such as the stimulus pole in an object localization experiment (Figure 4). The tests were for low correlation with the line detector, large left-right asymmetry in the image intensity about the detector, low mean intensity about the detector, and large angular change between steps. A corresponding user-adjustable threshold parameterizes each test. The tests are applied at each step as the curve is traced from the initiation point (Figure 4A,B). If one of these fails (Figure 4C,D), a number of single pixel steps are taken along the last trusted direction up to a user-settable maximum distance in an attempt to jump past the distrusted region (Figure 4E,F). Linear extrapolation is usually a good guess. If all tests are satisfied at one of the points, normal tracing is resumed (Figure 4G,H). Otherwise, the trace is terminated at the last trusted point (Figure 4G).

Occasionally, overlapping curves are generated during the tracing step. These need to be resolved before tracking so that individual whiskers have exactly one associated curve per frame. Searching for curves that cross the same pixel identifies pairs of potentially redundant curves. If all the points within an interval of one of the curves is within a small distance (typically 2 px) of the other curve and that interval consists of over 50% of the total length, then it is considered redundant. The shorter of the two curves is discarded; the shapes of shorter curves were not reliable enough to average. This procedure is repeated until there are no redundant curves left.

### Linking

Identifying individual whiskers is challenging. First, the tracing algorithm generates curves (false positives) for facial hairs, trimmed whiskers, or other artifacts in the scene (Figure 1C). The linking algorithm must correctly identify true whiskers against this noise. Second, the motion of whiskers can be difficult to reliably model [8], [12]. A typical trial may consist of slow, predictable motions interrupted by rapid transient motions that are occasionally too fast even for high speed (1000 fps) cameras to adequately sample [1]. Although difficult to track, these unpredictable motions may correspond to some of the most interesting data.

Our approach is based on the observation that whiskers are relatively long and maintain a consistent ordering along the face. We use a statistical model to describe the shapes and motions that are characteristic of whiskers. The model is then used to assign each traced whisker the most probable identity subject to the constraint that whiskers are ordered along the face.

For the majority of images, a simple length threshold can be used to remove false positives and leave exactly one traced curve per whisker. The linking algorithm is either supplied the number, *N*, of whiskers on the subject mouse, or estimates it as follows: For a given length threshold 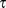, let 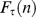 be the set of frames that have *n* curves longer than 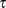. If *N* is not supplied, then we find 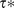 and 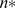 such that 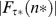 is maximal over all possible choices. In most of the data sets we have tested, 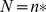, i.e. this heuristic is spot on. But when one or more whiskers frequently leave the field of view it may fail. If *N* is supplied, then we let 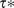 be the value for which 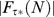 is maximal because the set of long curves in each frame in 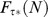 are always true whiskers. For the data sets tested here, 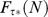 represented 99.8% of a video on average (min: 90.1%, max: 100%).

**Figure 5:**
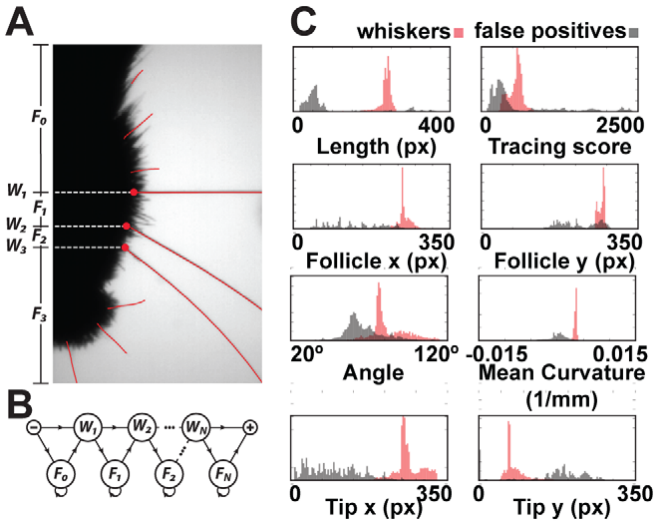
When linking *N* whiskers, each curve in a frame is assigned a label *W_1_* to *W_N_*, or *F_0_* to *F_N_*. Rules constrain the labeling to enforce consistent anterior-posterior ordering of whiskers. The most proximal point of curves labeled W_i_ or F_i_ must be posterior to that of curves labeled W_j_ or F_j_ when *i<j*, and at most one curve may be labeled *W_i_* for a given *i*. (A) A correct labeling is schematically illustrated. (B) These rules are encoded as transitions in a hidden Markov model. (C) Normalized feature histograms are used to compute the likelihood a curve is, or is not, a whisker.

The curves in the frames 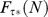 and their heuristic classification into whisker and non-whisker based on 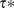 supplies a training data set for the specific video. From these, we can learn various characteristics to estimate the probability of a curve being one or the other. Specifically, we build normalized histograms, *n_f_*,*_W_* and *n_f_*,*_FP_* (Figure 5C), one for whiskers (*W*) and one for false positives (*FP*), for each of six features, *f*, using the training set. The features used are the angle near the face, average curvature, average tracing score, length, and endpoints of a curve. The angle and curvature are computed from a parametric third degree polynomial fit to each curve. These histograms are then used to estimate, 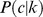, the probability that a curve *c* is of kind *k*, either *W* or *FP*, as the product,
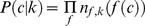
(1)To ensure no feature has zero likelihood, a count of one is added to each bin in a histogram before normalizing.

In (1) above, the features are assumed to be conditionally independent in order to simplify estimating feature distributions, even though this not strictly true in practice. Moreover, errors in the heuristic may introduce a systematic sampling bias. For example, at high deflection angles near the edge of the field of view, only a small segment of a whisker is imaged (Figure 1E, purple whisker). The traced curves from such segments might systematically fall below 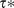 and be excluded from whisker feature distributions. As a result, estimated probabilities will be biased towards longer whiskers at milder deflection angles. Despite these caveats, the use of these feature distributions leads to a highly accurate result.

Appearance alone is not sufficient to uniquely identify individual whiskers in some cases. To address this, we designed a naive Bayes' classifier to determine the most probable identity of each traced curve subject to ordering constraints. The traced curves are ordered into a sequence, *C*, according to their time and relative anterior-posterior position. Identifying a curve, *c*, involves assigning a particular label, *l*, from the following set of *2N+1* labels. There is a label for each whisker, *W_1_* to *W_N_*, and labels, *F_0_* to *F_N_*, where *F_i_* identifies all false positives between whisker *W_i_* and *W_i+1_* (Figure 5A). The kind of a label, *K(l)*, is *W* if *l* is a whisker label, and *FP* if *l* is a false positive label. A curve labeled *W_i_* or *F_i_* must be posterior to curves labeled *W_j_* or *F_j_* for all *i<j*. For a given *i*, only one curve per frame may be labeled *W_i_*, but several curves may be labeled *F_i_*.

Applying Bayes' theorem, the most probable sequence of labels, 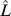, is given by
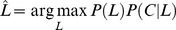
(2)where 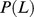 is the *a priori* probability of a labeling sequence, and 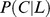 is the likelihood that the curves in *C* are generated from the sequence of items labeled by *L*. By design, certain labeling sequences are ruled out *a priori*. Conceptually drawing a directed edge between one identity and the possible identities of the next curve yields a directed graph (Figure 5B). For a video with *T* frames,
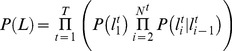
(3)where *N^t^* is the number of curves traced in frame *t*, and 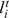 is the label of the *i*'th curve found in frame *t*. Values for 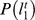 and 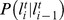 are estimated by computing the frequency of observed label pairs that occur in the a training set, 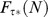. This describes a hidden Markov model for labeling curves in a single video frame. The optimal labeling can be computed efficiently with well-known dynamic programming techniques [21].

The likelihood, 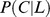, is computed under the simplifying assumption that the likelihood of a single curve depends only on the curves in the previous or following frame. Using this and applying Bayes' theorem,
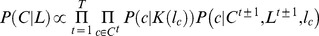
(4)where 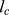 is the label *L* assigns to the curve *c*, *C^t^* is the (possibly empty) set of curves found in frame *t*, and 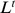 are the labels assigned to the curves in *C^t^*. The first component, 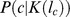, is the likelihood that *c* is an object of the kind denoted by label *l* which we estimate with formula (1).

The second component of (4), 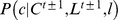, is interpreted as the likelihood that a curve is part of the same trajectory as corresponding curves in the previous (or following) frame. Similar to the approach used in equation (1) for estimating 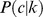, we need normalized histograms 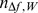 and 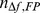 of the changes of whiskers and false positives between successive frames for each feature *f* over a “training” data set in which the corresponding curves in successive frames is known. While we could use the implied assignment over 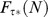, we first estimate a hopefully better assignment by restricting the model in (4) to use shape features alone. That is, 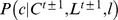 is treated as a constant and thus the assignment to labels in a frame can be computed independently of other frames. We then use this preliminary labeling over the frames of 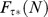 as the training set over which to build 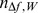 and 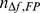.

Given these change histograms, one can estimate the correspondence likelihood according to the formula,
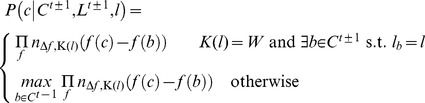
(5)Note that when evaluating this likelihood, a unique corresponding curve is not always present in the previous (or following) frame. There may be zero or many false positive curves in the previous frame with the same label. Similarly a whisker may be missing because it exited the field of view.

Directly solving for the most probable labeling (2) is complicated by the fact that the likelihood of a labeling in one frame depends on the labeling in neighboring frames. Our approach is to initially score each frame by computing 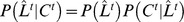, where 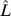 is obtained using shape features alone. In decreasing order of this score, we ‘visit’ the next best frame, say *t*, and update the label assignment for each of the two adjacent frames that maximizes the full version of (4), provided the adjacent frame has not already been visited. The new assignment replaces the current assignment and the frame's visitation order is updated according to the score of this new assignment (under the full model of (4)). In this way, we let the most confident frames influence their neighbors in a transitive fashion until every frame has been visited. This was critical for achieving high accuracy. Previous whisker tracking algorithms have relied on propagating the identity of a whisker frame-wise from the beginning of the video to the end, and as a result, an error in one frame is propagated throughout the rest of the video [11]–[13].

## Results/Discussion

Average processing time was 8 Mpx/s/cpu (35 fps for 640×352 pixel video) measured on a 2.6 GHz Intel Core2 Duo Macbook Pro with 2 GB of RAM, and was dominated by the CPU time required to trace curves; linking time was 0.5 ms per frame. This is faster than published speeds of similar whisker analysis packages (typically, 1–5 fps.) [11], [13], [15]. However, performance can be difficult to compare as implementations may improve over time. For example, our software is equally fast as the current version of the only other publically available whisker tracker[13]. More importantly, our software can readily be run on inexpensive cluster nodes to process videos in parallel. This is not the case for whisker trackers that require supervision [13] or that depend on software with expensive licensing requirements (such as Matlab) [11]–[15].

Tracing was accurate to within 0.2 px as estimated by analyzing 39 hand-traced whiskers. Individual hand-tracings had an accuracy of 0.23±0.2 pixels when compared against consensus curves. Mouse whiskers (row C) were automatically traced in images with resolutions from 5 µm/px to 320 µm/px using default settings and in noisy, low-contrast images (Text S1). For the best results, it is important to minimize motion blur and uniformly illuminate the scene, although illumination inhomogeneities can be partially corrected with background subtraction. In contrast with methods that use Kalman filtering [13], traced curves are not biased by whisker dynamics. Additionally, tracing is faithful even when curves are not well approximated by low order polynomials, in contrast to published tracing methods [13]–[15]. Whiskers could be detected and traced in videos of freely behaving rats (Video S2) and mice (Video S3) with all whiskers intact.

Linking accuracy was measured against a hand-annotated set of videos selected by choosing 100 random trials from behavioral sessions of 4 mice (1.32 million frames) [1], [17]. The curated videos captured a range of behavior including protracted bouts (>1 s) of whisking (Video S4), multiple whiskers simultaneously contacting an object (a thin metal pole) (Video S4), and extremely fast motion (>10,000°/second) (Video S5). Of the 4.5 million traced whiskers, 130 were incorrectly identified or not detected, less than one mistake per behavioral trial on average. Linking is robust to whiskers that occasionally leave the field of view (Video S6), and works well even at relatively low frame rates (100 fps; see Text S1). Lesser image quality will ultimately degrade results.

There are two principle sources of error in the linking. First, covariance between different features is ignored. For example, when the field of view is small, whiskers at high deflection angles might not be fully contained within the image. Any curve tracing one of these whiskers would appear shorter than the full whisker length. Under these conditions there is a strong correlation between angle and whisker length. The current model penalizes the shorter length because it is rarely observed; accounting for the covariance should eliminate this problem. Second, the estimated feature distributions are affected by systematic biases introduced from the heuristic used to initially guess whisker identity. A heuristic relying on a length threshold, like the one used here, may systematically reject these short curves at high deflection angle. This will result in a bias against strongly deflected whiskers.

Fundamentally, our linking method is applicable only to single rows of whiskers. The whiskers in videos imaging multiple rows of whiskers will be traced, but, because whisker images no longer maintain a strict anterior-posterior ordering, the linking will likely fail. Although this software was validated with images of one side of the mouse's whiskers, we have successfully tracked whiskers in images containing both whisker fields (Video S7). Also, the whiskers of freely moving rats and mice can be traced (Videos S2,S3). This suggests that it will be possible to use this software for two-dimensional tracking of single rows of whiskers in freely moving animals by incorporating head tracking [2], [11], [15], though we have not explicitly tested this.

**Figure 6:**
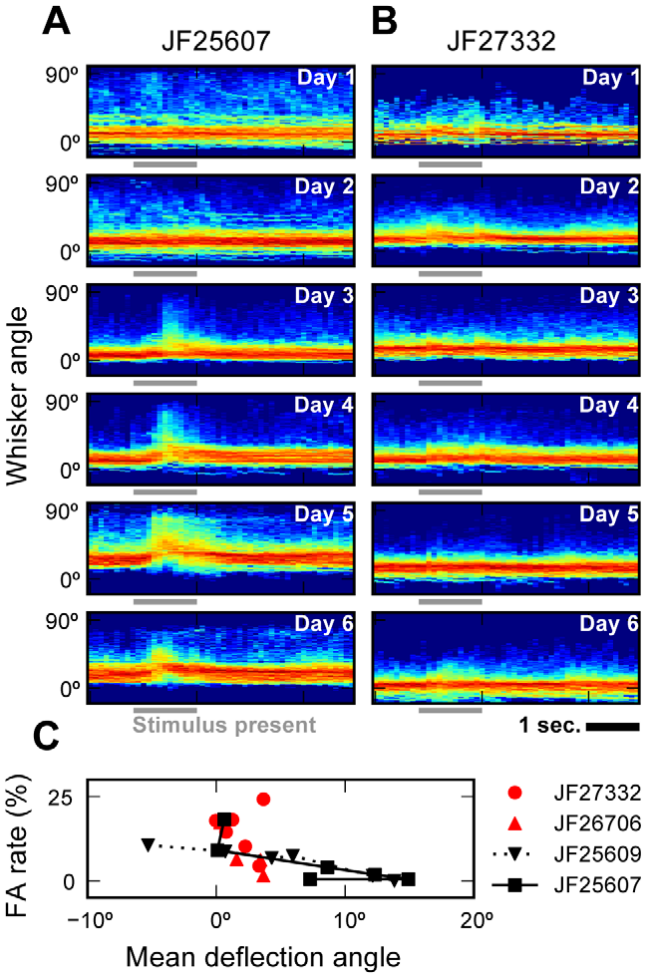
Changes in whisker behavior as mice successfully learn the object detection task. (A–B) Normalized histograms of whisker angle (1° resolution, whisker C1, log scale, 0° lies on the lateral axis) were computed over 100 ms time bins during the first 6 training sessions over correct rejection trials. Each histogram shows data from 21–150 trials. (A) Some mice, such as JF25607, increase the frequency of large deflections during stimulus presentation (trial counts: 99, 150, 135, 109, 102, and 90 respectively). (B) Others, such as JF27332, do not (trial counts: 21, 96, 90, 109, 107 and 86 respectively). (C) During stimulus presentation, an increase in mean deflection angle was correlated with a decrease in the false alarm (FA) rate, a measure of behavioral performance, for two mice (JF25609, JF25607). Two mice did not exhibit this correlation (R^2^: 0.14, 0.61, 0.97, 0.84 for JF27332, JF26706, JF25609, and JF25607 respectively).

We analyzed data acquired during learning of an object detection task (see Text S1) [17]. We tracked whiskers from four mice during the first 6 days of training (8148 trials, 3122 frames each). This allowed us to observe the changes in whisking behavior that emerge during learning of the task. We looked specifically at correct rejection trials (2485 trials), which were not perturbed by contact between object and whisker, to analyze the distribution of whisking patterns during learning. We characterized whisking by computing normalized histograms of whisker angle (1° resolution) over time (100 ms bins) for a single representative whisker (C1). The angle and position of other whiskers were strongly correlated throughout (r^2^>0.95) in these trials. Two patterns emerged. After learning, two mice (JF25607 and JF25609) showed changes in whisking amplitude and mean angular position when the stimulus was present (Figure 6A). The other two mice (JF27332 and JF26706) did not show nearly as much stereotypy and appeared to rely on lower amplitude whisking (Figure 6B). All mice held their whiskers in a non-resting anterior position during most of the trial [1]. For JF25607 and JF25609, reduction in task error rate was correlated with an increase in mean deflection angle during stimulus presentation (r^2^ = 0.97,0.84 respectively) (Figure 6C). This correlation was smaller for the other two mice, which also learned the task but appeared to rely on a different whisking strategy.

The forces at the base of the whisker constitute the information available to an animal for somatosensory perception. These forces are proportional to changes in whisker curvature [16]. The instantaneous moment acting at a point on the whisker is proportional to the curvature change at that point. In contrast to whisker tracing software that represents whiskers as line segments[14], [15] or non-parametric cubic polynomials[13], the curves produced by our tracing algorithm provide a high-resolution representation of whisker shape, allowing measurement of varying curvature along the length of the whisker.

**Figure 7:**
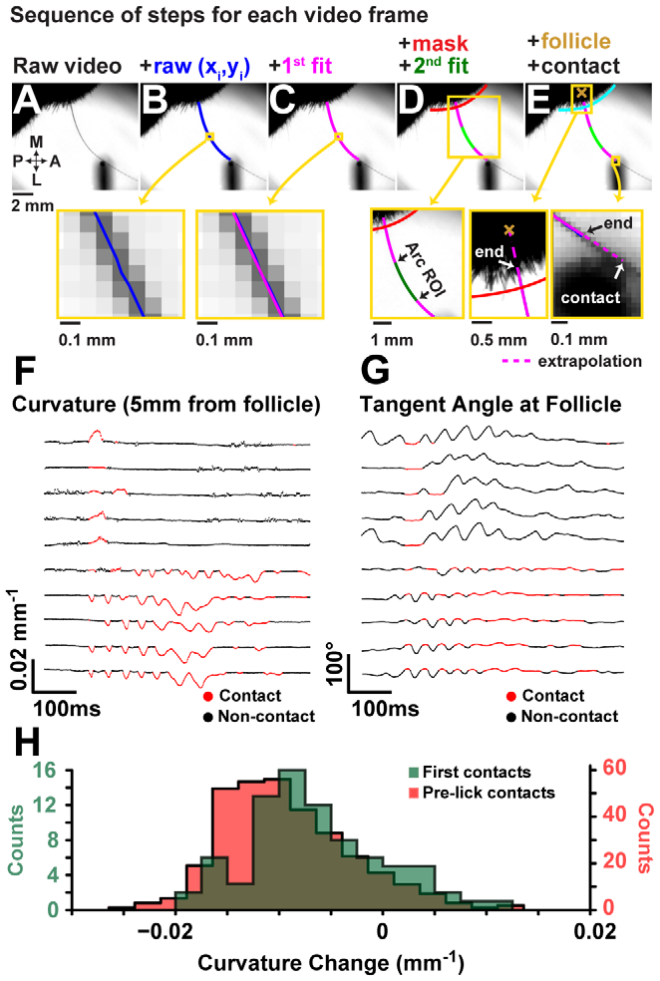
Analysis of curvature change on contact. (A–E) The sequence of steps used to extract detailed curvature measurements. (A) Whiskers in raw video frames are automatically traced and linked, yielding (B) an identified set of curves for each frame. (C) The raw curve is fit with a 5^th^-degree parametric polynomial. (D) A mask is specified to determine where the curve intersects the face. Within a small interval (1–2.5 mm path length) about an interest point chosen for high signal to noise, the raw curve is re-fit to ensure measurements are not biased by whisker shape outside the interval. This new fit is to a 2^nd^-degree polynomial. The curvature at the interest point is then measured as the curvature of this 2^nd^ fitted curve. (E) Follicle position is estimated by extrapolating a fixed distance into the face from the mask. Similarly, curves are extrapolated, when necessary, to contact points on the pole. Trajectories for curvature (F) and the angle of the whisker at its base (G) are shown for the first contacting whisker in 10 trials grouped by whether the first contact was during a retraction (top 5) or protraction (bottom 5). Trajectories are aligned to first contact. The intervals when the whisker is in whisker-pole contact are highlighted in red. (H) Histograms of peak contact curvature change (from resting) for the first whisker-pole contact in each trial (green) and all whisker-pole contacts prior to an answer-lick (red).

To illustrate this feature we measured curvature in the following manner: For each video frame (Figure 7A), the traced whisker midline (Figure 7B) was fit via least-squares to a parametric 5^th^ degree polynomial of the form:
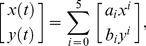
(6)where [*x(t),y(t)*] are points that span the curve over the interval 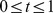, and *a_i_,b_i_* are the fit parameters (Figure 7C). Curvature was measured at a specified distance along the curve from the follicle chosen for best signal to noise. The follicle was not imaged, and so the intersection between the curve and an arc circumscribing the face was used as a reference point instead. The curvature was then measured as the mean curvature inside a 1–2.5 mm long region about the point of interest where it was approximately constant. To ensure the measurement was not biased by shape outside the interval, another parametric polynomial fit (degree 2) was performed, but over the region of interest rather than the entire curve (Figure 7D). The follicle position was set by linearly extrapolating a set distance into the face. The point of whisker-pole contact was determined as the closest point to the center of the pole on the curve or on a line extrapolated from the nearest end of the curve (Figure 7E).

Time series of curvature measurement (Figure 7F, measurements at 5 mm from follicle) can be used to identify most contact events. For whisker-pole contacts first made during a protraction, the first touch tends to be followed by a series of more forceful contacts. Contacts are correlated with relatively constant angular position of the whisker (Figure 7G). This indicates the mouse may press the whisker against the pole by translating the whisker pad rather than by rotating the whisker. The distribution of peak curvature-change during a whisker-pole contact indicates more the bulk of contacts were made during protraction and that these were more forceful (Figure 7H).

### Availability

The software and documentation are freely available online (http://whiskertracking.janelia.org) as cross-platform (Windows, OS X, Linux) source code written in C with Python and Matlab interfaces included. Pre-built binaries are also available. A graphical user interface is provided to aid semi-automated tracing as well as the viewing and editing of results. The software is capable of analyzing 8-bit grayscale StreamPix (Norpix Sequence Format: SEQ), TIFF, MPEG, MOV, and AVI formatted video.

## Supporting Information

Dataset S1 Sample data and tutorial.
(ZIP)

Figure S1 Line bias correction. (A) Raw data exhibits a fixed-pattern artifact where odd-numbered lines are systematically darker than bright lines. (B) This artifact is corrected by multiplying odd-lines by a factor estimated from the raw data itself. This removes the stripes without blurring or reducing the contrast of whiskers. Scale bar, 0.5 mm.
(TIF)

Text S1 Methodological details.
(DOC)

Video S1 Video overlaid with high scoring seeds (eccentricity>0.99) for each frame. Pixels are colored according to the orientation indicated by the seed.
(MP4)

Video S2 Results of automatic tracing applied to video 500 Hz video (1.7 s duration) of a freely behaving rat exploring a corner with its full whisker field. The video was obtained with permission from the BIOTACT Whisker Tracking Benchmark [22] (Clip ID:behavingFullContact, courtesy of Igor Perkon and Goren Gordon). It has been cropped, and the frame rate has been changed for presentation.
(MP4)

Video S3 Results of automatic tracing applied to a video acquired at 100 Hz video (8 s duration) of a freely behaving mouse with its full whisker field. Background subtraction was performed before tracing to reduce the effects of non-uniform illumination. Because no image of the scene without the animal present was available, the background was estimated for each pixel as the maximum intensity observed at that point throughout the video. The video was obtained with permission from the BIOTACT Whisker Tracking Benchmark [22] (Clip ID: behavingMouse100, courtesy of Ehud Fonio and Ehud Ahissar). It has been cropped, and the frame rate has been changed for presentation.
(MP4)

Video S4 Results from a full trial. The results of automated tracing and linking applied to a video (500 Hz, 9.2 s duration) of a head fixed mouse trimmed to a single row of 4 whiskers interacting with a pole. Curves that were classified as whiskers are colored according to their identity, and otherwise they are not shown. Multiple whiskers simultaneously interact with the pole at 1.2–1.4 sec into the trial. Protracted bouts of whisking can be observed throughout the video.
(MP4)

Video S5 Tracing and linking is robust to rapid changes in whisker angle. The results of automated tracing and linking applied to a video (500 Hz, 150 ms) of a head fixed mouse trimmed to a single row of 3 whiskers interacting with a pole. Curves that were classified as whiskers are colored according to their identity, and otherwise they are not shown. One whisker (red) has been trimmed so it cannot contact the pole. The green whisker presses against the pole, and quickly flicks past it as it is removed from the field. This is the fastest angular motion (16°/ms) observed in the data set used to measure tracking accuracy.
(MP4)

Video S6 Linking is robust to whiskers that leave the field of view. The results of automated tracing and linking applied to a video (500 Hz, 190 ms) of a head fixed mouse trimmed to a single row of 3 whiskers interacting with a pole. Curves that were classified as whiskers are colored according to their identity, and otherwise they are not shown. Two whiskers (green and blue) are frequently occluded by the lick-port (black bar, lower right), but they are properly identified before and after such events.
(MP4)

Video S7 Tracing and linking of whiskers bilaterally. The results of automated tracing and linking applied to a video (500 Hz, 1 s duration) of a bilateral view to a head fixed mouse trimmed to a single row of whiskers (2 on each side).
(MP4)
