# Supplementary material for: Automated Tracking of Whiskers in Videos of Head Fixed Rodents
Source: PLoS Comput Biol. 2012 Jul 5;8(7):e1002591. doi: 10.1371/journal.pcbi.1002591 (PMC3390361; doi:10.1371/journal.pcbi.1002591)

Installation and Tutorial Instructions

Installation

For the most up-to-date and detailed documentation see http://whiskertracking.janelia.org.

Obtaining the software

There are two ways to install. It's simplest to use one of the installers found here. If one isn't available for your system, you will need to install by building the source code available here. The source code builds and has been tested on Ubuntu 10.10, Fedora 10, Windows XP, Windows 7, and OS X 10.5 and 10.6 on both 32 and 64-bit machines. Instructions for building may be found in the README file located in the root directory of the repository.

Installation

These instructions cover use of one of the pre-built installers. These will install the command line tools that are central to the automated video analysis as well as development libraries, python extensions, matlab functions, and the graphical user interface.

Windows

1. Download and run the appropriate installer for your system.
2. In the installation wizard, choose to install Everything when you get the chance. Otherwise, accept all defaults.
3. The software will get installed to
   `C:\Program Files (x86)\WhiskerTracking`
   or
   `C:\Program Files\WhiskerTracking`

   In that folder, the following subdirectories should be populated:

- `bin`
- `lib`
- `matlab`
- `python`
- `ui`

4. (Optional) Add the `bin` subdirectory to your environment's PATH variable.

OS X and Linux

1. Download and unarchive the appropriate pre-built binary package.
2. Unarchiving should populate a directory tree in the destination folder that looks like:
    - `bin`
        - `whisk`
            - `ui`
    - `lib`
        - `whisk`
    - `share`
        - `whisk`
            - `python`
            - `matlab`
3. (Optional) Add the `bin` subdirectory to your environment's PATH variable.

(Optional) Install the Python Environment

Several Python dependencies are required to use the included graphical user interface and python extensions. Install the following in order:

1. Python 2.6
2. numpy 1.4.1
3. scipy 0.8
4. matplotlib 0.99.3
5. pygame 1.9
6. aggdraw 1.2a3

Refer to each package's documentation to find the best way to install for your system.

(Optional) Install and configure Matlab

Refer to Matlab's (http://www.mathworks.com) documentation for installation instructions. Matlab is not required for analyzing video or viewing results. The included functions have been tested on both 32 and 64-bit versions of Matlab (R2010 and later).

To use the included Matlab functions, they should be added to your matlab path (see the addpath function in Matlab). The actual path to add vary's between operating sytems.

Windows (example)

```
>> addpath('C:\Programs Files (x86)\WhiskerTracking\matlab');
```

Mac OS X and Linux (example)

```
>> addpath('/path/to/WhiskerTracking/share/matlab');
```

Sample Data: Automatic Whisker Tracking

Download the sample video here.

For this tutorial, it is assumed you understand how to call executables from the command line in your operating system of choice. Additionally, it is assumed that the whisker tracking `bin` directory has been added to the `PATH` variable for your environment.

Open a command prompt and change directory to where the sample video is stored.

1. Trace whiskers. Run:

   ```
   % trace sample.mp4 sample.whiskers
   ```

   The output may look something like this:

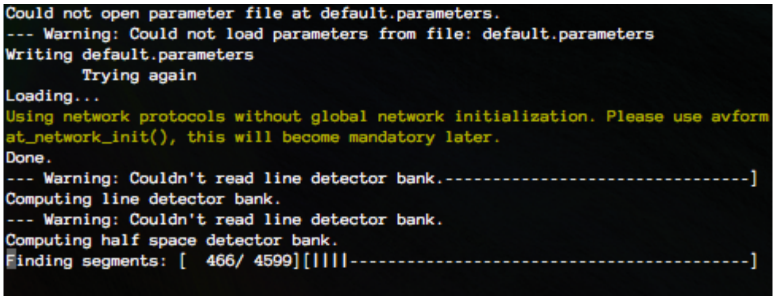

```
Could not open parameter file at default.parameters.
--- Warning: Could not load parameters from file: default.parameters
Writing default.parameters
        Trying again
Loading...
Using network protocols without global network initialization. Please use avform
at_network_init(), this will become mandatory later.
Done.
--- Warning: Couldn't read line detector bank.-------------------------------]
Computing line detector bank.
--- Warning: Couldn't read line detector bank.
Computing half space detector bank.
Finding segments: [  466/ 4599][||||----------------------------------------]
```

The `default.parameters` file is generated on the first run as well as the "line detector bank" and "half line detector bank." On subsequent runs from the same directory, these files will be reused. The `default.parameters` file contains a documented list of the parameters used by the software. They rarely need to be changed, but they can be by editing this file.

This is the most time consuming step. It should take a few minutes to run.

2. Generate measurements of the traced shapes. These are used in later steps to determine the correspondance between traced objects and whiskers. From the command line, run:

```
%  measure --face left sample.whiskers sample.measurements
```

In this video the face is on the left side of the frame. Knowing where the face is tells measure which side of traced objects should be considered the follicle.

3. Usually, the tracing step identifies too many objects (e.g. facial hairs are often traced). Use `classify` to generate a first guess of which traced shapes are actually whiskers. Here, the strategy is to be conservative. It's ok to identify some whisker shapes as "not a whisker," but identifying, for example, facial hairs as whiskers is more difficult to tolerate in later steps. Run `classify` from the command line:

```
% classify sample.measurements sample.measurements left --px2um 0.04 –n -1
```

The same file is used as the source and destination file. Again, the face is

specified to be on the left side of the image. A length threshold is used to discriminate between traced whisker segments and other traced shapes; the `px2mm` parameter provides the spatial scale. Finally, we specify that we should attempt to automatically determine the number of whiskers in the field throughout most of the video. See `classify --help` for more options.

4. Using the results from the previous step, identify which traced objects belong to which whiskers throughout the video. From the command line, run:

```
% reclassify sample.measurements sample.measurements
```

Again, the same file is used as input and output.

View the results

There are several ways to browse the results.

Python GUI

The Python dependencies must be installed.

From the command line run:

```
% python path/to/whiskertracker/ui/ui2.py path/to/sample.mp4
```

The python script will attempt to find the relevant `sample.whiskers` and `sample.measurements` files based on the location of `sample.mp4`. If they are not found, try appending the file names to the command line.

This will open a window that looks like:

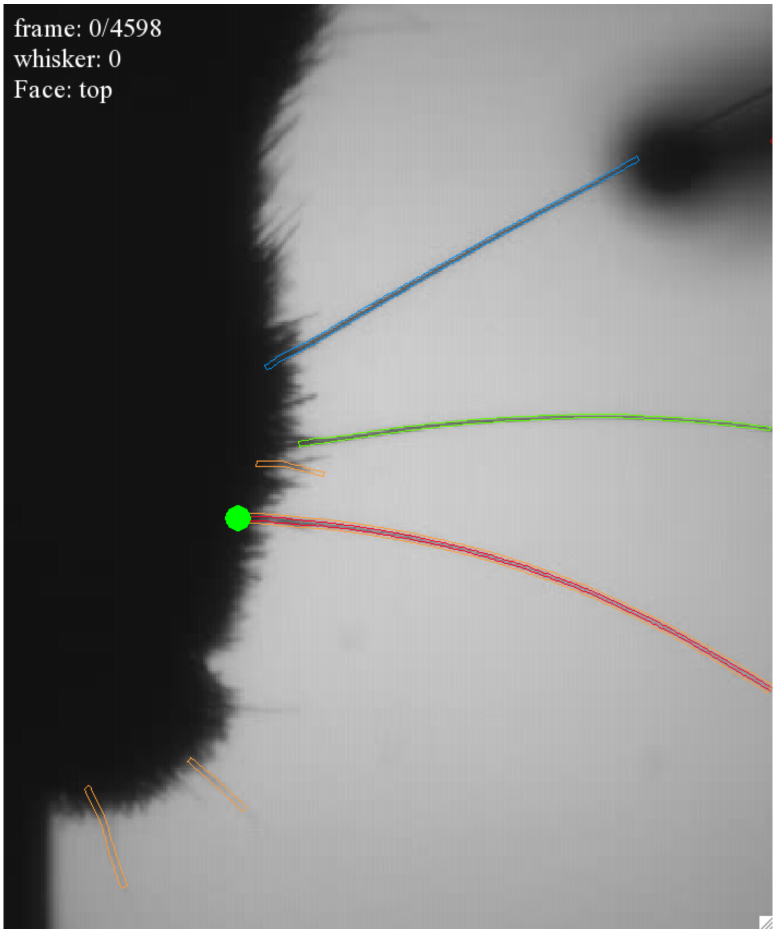

Refer to the `README` in the whisker tracking `ui` directory for keyboard controls. Use the left and right arrow keys to browse through video frames.

Matlab

The following code snippet loads tracking data from a file called `trial.measurements` and plots whisker angle over time for all the tracked whiskers.

```
%% Load the file
measurements = LoadMeasurements('trial.measurements')

%% Set the frame rate
dt = 0.002; %seconds per frame

%% Get the data and plot
%  Note: Be careful about the data types returned in the measurements struct.
%        They're not all doubles.

time  = double([measurements(:).fid]) .* dt; % time in seconds
angle = [measurements(:).angle];             % angles in degrees
colors = ['r','g','b','k','c','m'];          % add more colors as necessary
clf();
hold on;
for whisker_id = 0:max([measurements(:).label])
  mask = [measurements(:).label]==whisker_id;
  plot(time(mask),angle(mask),colors(whisker_id+1));
end
xlabel('Time (s)');
ylabel('Angle (deg.)');
hold off;
```

This will generate a plot like:

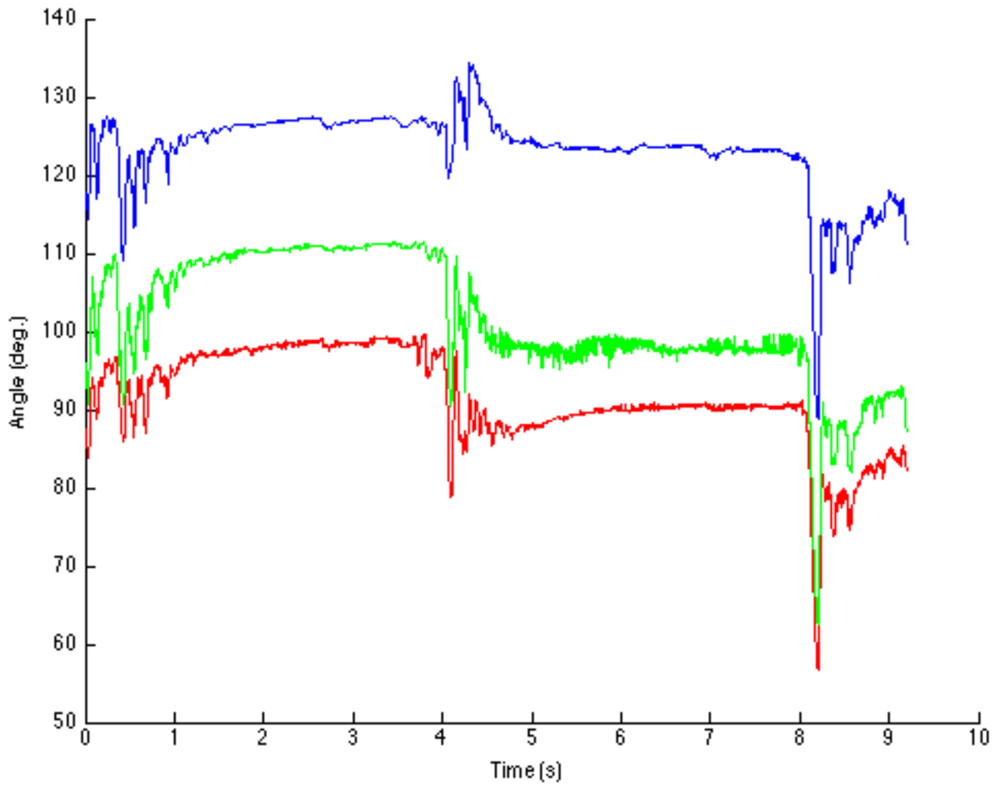

Python

This example requires [matplotlib (pylab)] in addition to the other Python dependencies listed in the Installation instructions.

The following code snippet loads tracking data from a file called `trial.measurements` and plots whisker angle over time for all the tracked whiskers.

```
import traj
from numpy import *
from pylab import *

data = traj.MeasurementsTable('trial.measurements').asarray()

dt = 0.002 # seconds per frame

for i in xrange(data[:,0].max()+1):
  mask = data[:,0]==i
  plot(data[mask,1]*dt,data[mask,5])
xlabel('Time (s)')
ylabel('Angle (deg.)')
show()
```

This will generate a plot like:

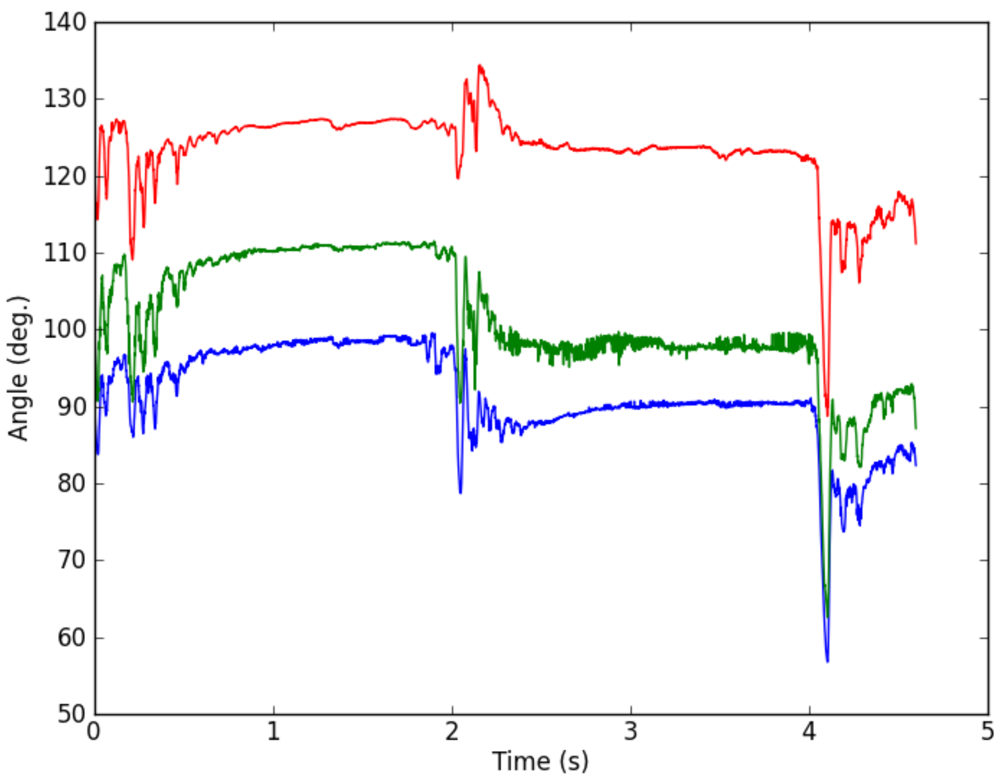

Supplement: Dataset S1 — Sample data and tutorial. (ZIP) [file pcbi.1002591.s001.zip › SampleDataAndTutorial/Sample Data and Tutorial.pdf]
