# Supplementary figures and images for: Automated Tracking of Whiskers in Videos of Head Fixed Rodents
Source: PLoS Comput Biol. 2012 Jul 5;8(7):e1002591. doi: 10.1371/journal.pcbi.1002591 (PMC3390361; doi:10.1371/journal.pcbi.1002591)

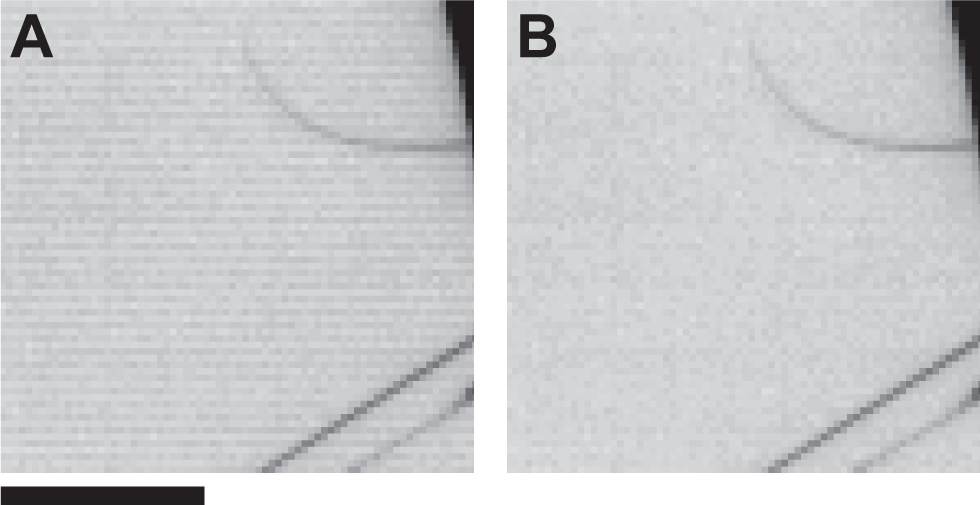

Supplement: Figure S1 — Line bias correction. (A) Raw data exhibits a fixed-pattern artifact where odd-numbered lines are systematically darker than bright lines. (B) This artifact is corrected by multiplying odd-lines by a factor estimated from the raw data itself. This removes the stripes without blurring or reducing the contrast of whiskers. Scale bar, 0.5 mm. (TIF) [file pcbi.1002591.s002.tif]
