# Supplementary material for: Automated Tracking of Whiskers in Videos of Head Fixed Rodents
Source: PLoS Comput Biol. 2012 Jul 5;8(7):e1002591. doi: 10.1371/journal.pcbi.1002591 (PMC3390361; doi:10.1371/journal.pcbi.1002591)
Supplement: Text S1 — Methodological details. (DOC) [file pcbi.1002591.s003.doc]

# Automated tracking of whiskers in videos of head fixed mice.

## Supplementary Text S1

### Apparatus

The apparatus and behavioral task were similar to one described previously [1]. Briefly, a stainless steel dowel pin (the “pole”), acting as a stimulus object, was driven in the anterior-posterior dimension by a motor. The pole was rapidly (0.5 s) moved in and out of the reach of whiskers during a trial by a pneumatically actuated slider (Figure 1A-B). Water rewards were delivered via a custom acrylic “lickport” positioned in front of a mouse within reach of the tongue. Neither the lickport nor the object were visible in high speed videos.

Whiskers were illuminated using a high power LED source (940 nm, Roithner) and condenser optics (Thorlabs). Images were acquired through a telecentric lens (0.36X, Edmund Optics) by a high-speed CMOS camera (EoSense CL, Mikrotron, Germany) running at 500 frames/sec (640 x 352 pixels; resolution, 42 pixels/mm). Image acquisition was controlled by Streampix 3 (Norpix, Canada).

### Behavioral Task

Adult male C57BL/6Crl mice (Charles River) were used according to protocols approved by the Janelia Farm Institutional Animal Care and Use Committee. Whiskers were trimmed to a single row (row C). We trained head-fixed mice in a Go/No Go whisker-based, active object detection task [1]. Following a sound cue, a pole moved to one of several target positions within reach of the whiskers (the “Go stimulus”) or to an out-of-reach distracter position (the “No Go stimulus”). In each trial, the pole was presented at a single location. Target and distracter locations were arranged along the anterior-posterior axis; the distracter position was most anterior. Mice reported the presence of the pole within a target position or in a distracter position by licking or withholding licking, respectively. Licking on Go trials (Hits) was rewarded with water, whereas licking on No Go trials (False Alarms) was punished with a time-out. Trials without licking (No Go, Correct Rejection; Go, Miss) were not rewarded or punished. All mice showed significant learning within the first 2-3 sessions (one-tailed bootstrap test, p<0.001). Subsequently performance improved, reaching expert level after 3-6 training sessions (approximately 90 % correct, p<0.001).

### Preprocessing

The high-speed CMOS cameras used to acquire data [1] exhibited a systematic striped pattern (Figure S1A). Analysis indicated the proportional light response of odd scan lines differed from even scan lines. To correct for this multiplicative bias (2-3%), the average ratio of intensities between a pixel and the pixel directly above it was computed over pixels in odd scan lines. To avoid saturation and quantization errors, only pixels with intensities above the image mean (150-160 typically for 8-bit pixels) and 2% below saturation (250) were considered. To correct the image, evenly numbered lines were multiplied by the measured bias, visibly improving the image (Figure S1B).

### Tracing and Linking accuracy

We quantified detection errors and the linking accuracy of this software over a data set consisting of 1.32 million curated video frames comprising 4.5 million whisker traces. Production data sets may be somewhat larger. Over such large data sets, manually verifying the output of automated tracking frame by frame is extremely time consuming. Our aim was to achieve accuracy sufficient to obviate the need for extensive manual curation. This is achieved by, first, a linking accuracy better than 1 mistake per thousand images. Second, the algorithm outputs a per-frame confidence score that may be used to identify potentially error prone frames. Third, rare errors can be detected as discontinuities in whisker trajectories.

Videos were curated by hand with machine assistance to assess the linking accuracy. Work was divided among 5 experts who performed the curation over a period of 1 month. In all, 400 videos were annotated (an average of 4 videos per person per day with 3300 frames per video). Since rare events can dramatically affect accuracy, it is important to validate over a large data set. Accuracy was computed as the fraction of correctly labeled whisker-like curves. Curation proceeded as follows: whiskers midlines were traced automatically to generate an initial data set. Then, using a graphical interface (GUI) a human curator would label individual whiskers by clicking on a curve. Under user supervision, a click could be automatically propagated to the next frame, so that several curves could be labeled in rapid succession. Rarely, when the tracing result was missing or clearly incorrect, the curator would retrace the curve. Software was used to tabulate differences between the curated labels and the labels assigned after automated tracing and linking. Each difference was manually double-checked. Often the difference was an error made by the curator, but otherwise it was counted as an error. Intervals where a puff of air was administered as a negative reward or where a paw obstructed the whiskers were excluded from this analysis. Of the 4.5 million annotated whiskers, 130 were incorrectly identified or not detected.

We analyzed linking performance at different frame rates by temporally down-sampling a sample video with fast motion and long periods of exploratory whisking (Video S4) from 1000 Hz to 4 Hz by factors of two. Linking performance remained constant (zero errors) at frame rates above 100 Hz. Below 100 Hz, whisker motion was such that the identity of the whiskers was sometimes confused. Ignoring frame-to-frame correspondence, so that only the shape and order of whiskers informed their identity, improved results at low frame rates (4-100 Hz). Since tracing is performed independently on each frame, the frame rate has no effect on the measured shapes of whiskers.

Tracing was, on average, accurate to within 0.2 pixels as determined by comparing to hand-traced whiskers. Five curators independently and manually traced 39 whiskers imaged at 40 µm resolution (one whisker per image). 14 of these involved contact with the stimulus object. Whiskers appeared 5-7 pixels wide near the follicle. Signal to noise (SNR) was typically greater than 10. Signal was measured as the absolute difference between the background intensity and the darkest intensity along the whisker backbone. Noise was estimated as the standard deviation of the background intensity.

For each whisker the start and end point were chosen, and the whisker backbone was located for 100 equally spaced intermediate points between the whisker base and an end-point by clicking on a sub-pixel position. The end-point was chosen to be either the last visible point at the tip of the whisker or, for whisker contacting the pole, the point of contact. The hand tracings were then averaged. This consensus tracing served as the ground truth. The smallest distance between each point and the automatically traced curve was then computed. This represents an upper bound on the accuracy of positions estimated during tracing. Hand traced curves could not be distinguished from the automated tracing result; the average hand-tracing accuracy was 0.23 ± 0.2 pixels. Tracing accuracy estimates differed by less than 1% between the 39 whiskers indicating these results should be representative of similar images.

Whiskers can be traced in images with SNR>3 using default settings. This was evaluated both by adding Gaussian noise to an existing image, and also by measuring the SNR around low-contrast whisker tips throughout the data set. Tracing was evaluated at different pixel resolutions by first resampling a target image acquired with 40 µm/px resolution to each of the test resolutions (5,10,20,80,160,320 µm/px) using bicubic interpolation, and then tracing whiskers in each image using default parameters. Tracing was also robust to video compression (MPEG4, compression ratio 0.03).

This measure does not reflect how accurately curve endpoints were determined. Endpoint determination is more subjective. Follicles are hidden by facial hair, and often the distal tips of whiskers are blurred by motion or leave the field of view. Facial contrast, and the visual appearance of facial hair change precisely where the proximal end of a whisker is determined to be. In our data, follicle positions are determined with an accuracy of 8 px along the direction of the whisker using default tracing parameters.

## References

1. O'Connor DH, Clack NG, Huber D, Komiyama T, Myers EW, et al. (2010) Vibrissa-based object localization in head-fixed mice. J Neurosci 30: 1947–1967. doi:10.1523/JNEUROSCI.3762-09.2010.
